# Catheter ablation of premature ventricular contractions originating from aortic sinus cusps in a patient with dextrocardia and situs solitus: A case report

**DOI:** 10.1097/MD.0000000000008947

**Published:** 2017-12-01

**Authors:** Chao-Feng Chen, Xiao-Hua Liu, Xiao-Fei Gao, Bin Chen, Yi-Zhou Xu

**Affiliations:** Hangzhou First People's Hospital, Nanjing Medical University, Zhejiang Chinese Medical University, Shangcheng District, Hangzhou, Zhejiang, China.

**Keywords:** aortic sinus cusps, catheter ablation, dextrocardia, premature ventricular contractions

## Abstract

**Background:**

Premature ventricular contractions (PVCs) originating from aortic sinus cusps is not infrequent and can be eliminated effectively by radiofrequency ablation with rare complications. However, after a review of the medical literature, and to our knowledge, this is the first case of successful idiopathic aortic sinus cusps–PVC–ablation using a 3-dimensional (3D) mapping system in an adult with dextrocardia.

**Methods:**

A 62-year-old male with dextrocardia and situs inversus underwent catheter ablation of frequent PVCs. The electrocardiograms (ECG) were recorded by placement of the electrodes in reversed positions. The PVCs exhibited left bundle branch block and inferior axis QRS morphology with transition at leads V2–V3. The activation mapping indicated the earliest site of ventricular activation between the left and right aortic sinus cusps, highlighting that catheter ablation was successful at this point.

**Results:**

The catheter ablation was successful between the left and right aortic sinus cusps, and the PVCs were not detected for the subsequent 30 min following the procedure as well as for the rest of the hospital stay.

**Conclusion:**

Combined with ECG electrodes in reversed positions and 3D electroanatomical mapping system, catheter ablation of PVCs originating from aortic sinus cusps in patients with dextrocardia can be safely and effectively performed.

## 1 Introduction

Premature ventricular contractions (PVCs) originating from aortic sinus cusps are a common condition that can be eliminated successfully by ablation. The ablation procedure may be challenging to the medical practitioner due to the morphological changes of PVCs in patients with dextrocardia. The technique of 3-dimensional (3D) mapping can aid the success of the ablation procedure. Certain cases have been reported regarding PVCs that originated from the right ventricular outflow tract (ROVT) and were successfully eliminated in patients with dextrocardia.^[1]^ Nevertheless, a case of aortic sinus cusps–PVC–ablation using a 3D mapping system in a dextrocardia patient has not been reported to date.

## 2 Case report

**Figure 1:**
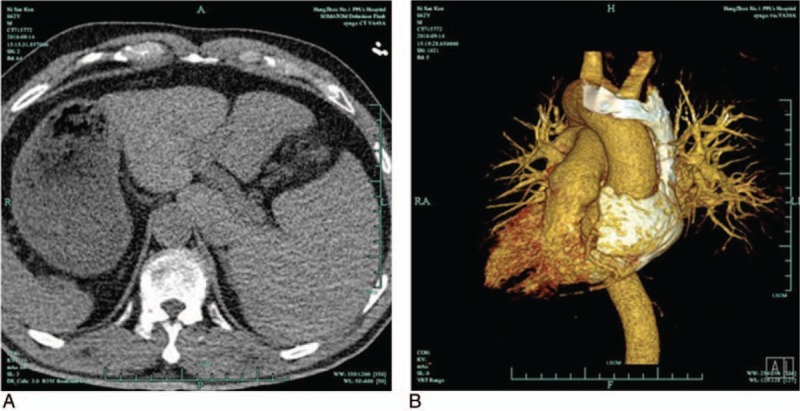
(A) Computerized tomography image of patient; (B) 3-dimensional computerized tomography reconstruction of patients.

**Figure 2:**
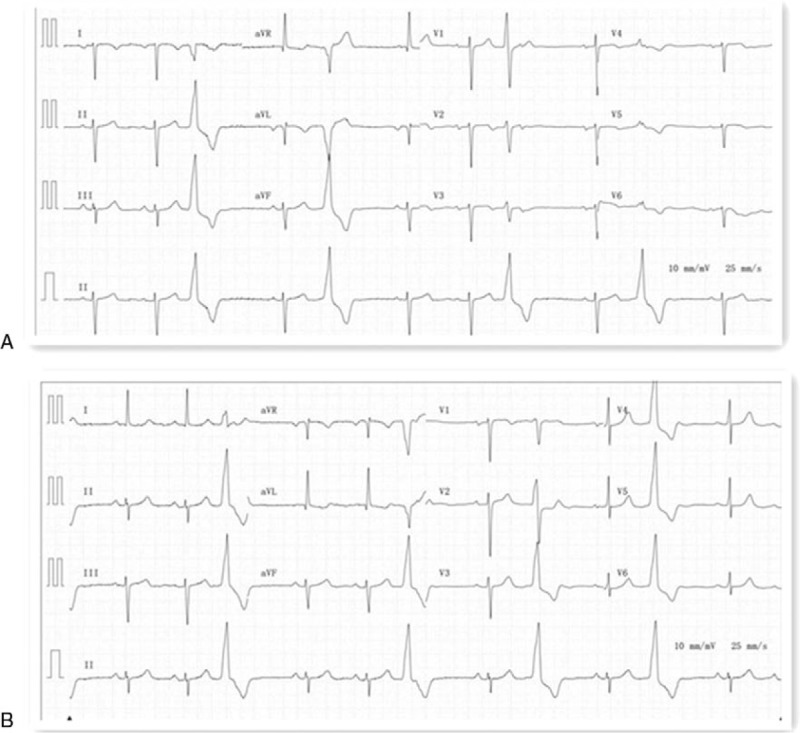
(A) Standard 12 ECG over the left chest; (B) 12 ECG with the precordial leads positioned over the right chest as a complete “mirror image” of the usual lead positions. ECG = electrocardiogram.

A 62-year-old man was referred for catheter ablation of idiopathic PVCs due to severe persistent palpitations for >1 year. The computerized tomography (CT) and 3D CT reconstruction demonstrated dextrocardia with situs inversus (Fig. 1A and B). The initial electrocardiogram (ECG) indicated an inversion of the electrical waves in I, aVR, and aVL and inferior axis with LBBB morphology (Fig. 2A). Subsequently, the ECG was recorded by reversed placement of the electrodes to the corresponding positions. PVCs exhibited a left bundle branch block (LBBB) and inferior axis QRS morphology with transition at leads V2–V3 (Fig. 2B). Echocardiography revealed a normal “left” ventricular ejection fraction (LVEF 58.5%) in the absence of structural abnormality. In addition, inflammatory markers of this patient were normal (Table 1).

**Table 1:**
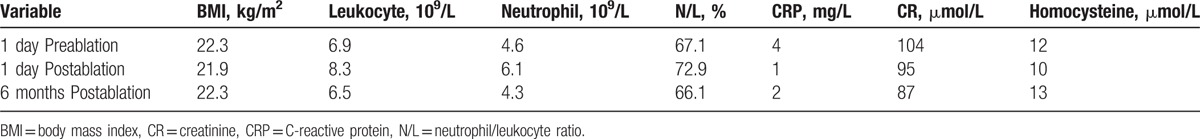
Baseline patient characteristics.

**Figure 3:**
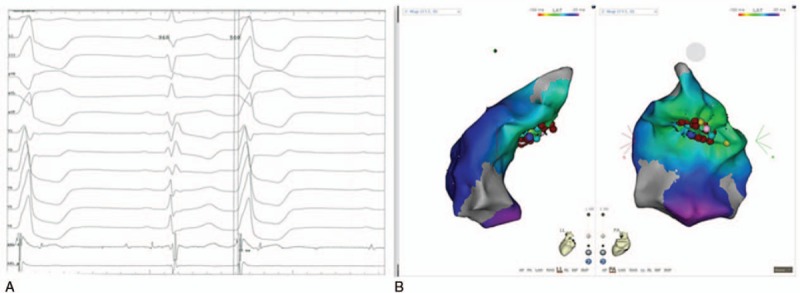
Cardiac tracings (A) and activation maps (B) indicating the earliest site of ventricular activation at the right ventricular septum.

The CARTO 3 electroanatomical mapping system was used to construct a detailed map of the ventricle. According to the morphology of PVCs, the possibility that the condition originates from the right ventricular septum cannot be dismissed. The activation mapping in the right ventricular septum revealed the earliest site of ventricular activation, 30 ms earlier than the QRS complex on the surface ECG (Fig. 3A). However, the catheter ablation was unsuccessful (Fig. 3B).

**Figure 4:**
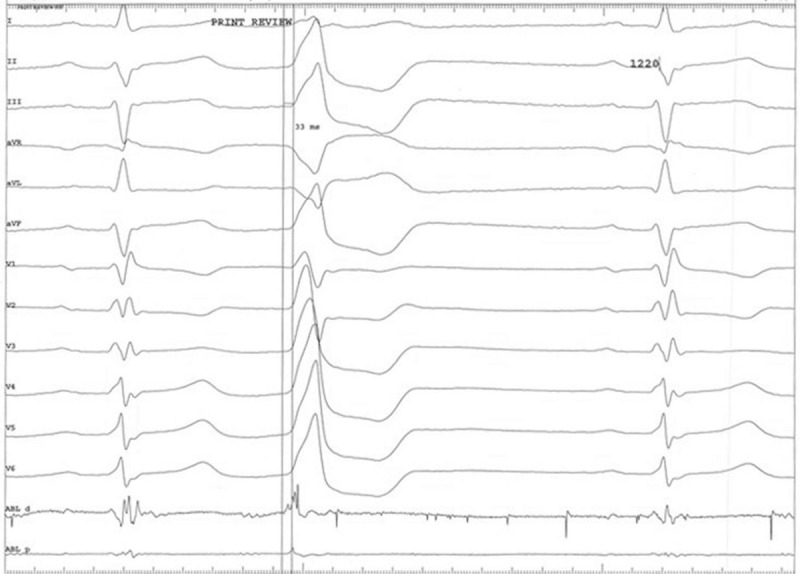
Cardiac tracings indicating the earliest site of ventricular activation between the left and right aortic sinus cusps.

**Figure 5:**
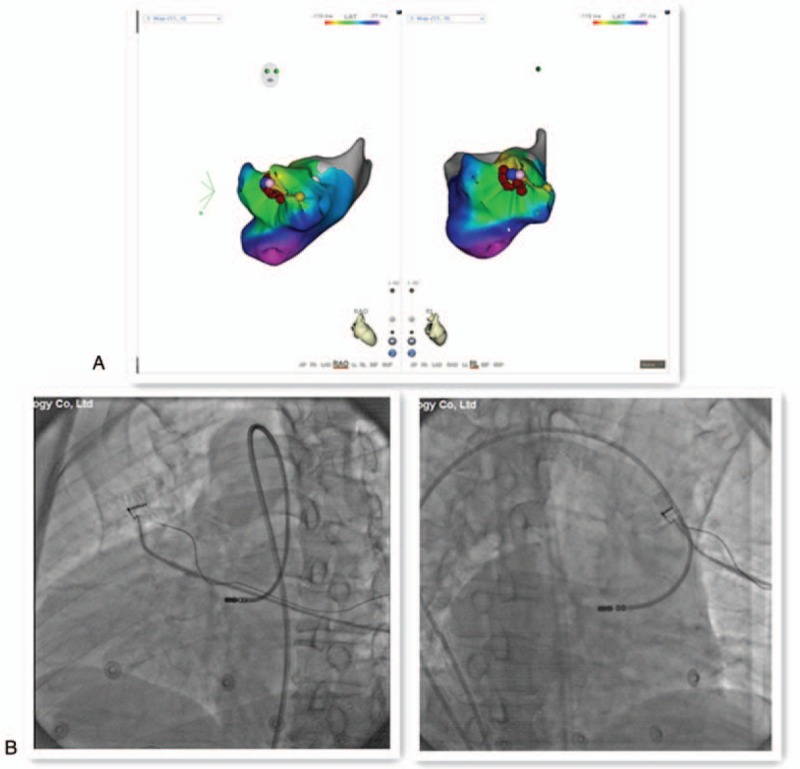
Activation maps (A) and fluoroscopic images (B) indicating the successful ablation site.

**Figure 6:**
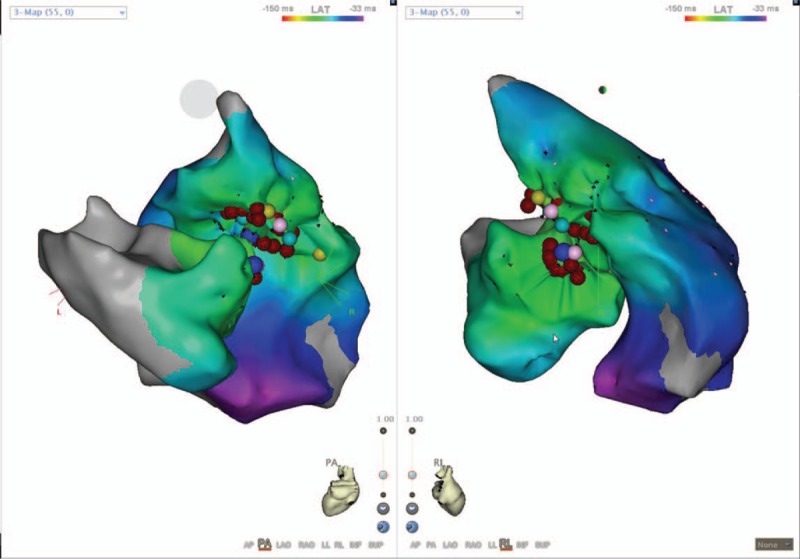
Integration of left and right ventricle models of CARTO 3.

Following activation mapping in the right ventricular septum, the mapping was conducted in the aortic sinus cusps via the right femoral artery. During PVCs, the local ventricular activation occurred at the earliest time point between the left and right aortic sinus cusps and was detected 33 ms earlier from the QRS complex on the surface ECG (Fig. 4). The catheter ablation was successful at the aforementioned point (Fig. 5A and B), whereas the PVCs could not be induced by ventricular stimulation. The PVCs were not detected for the subsequent 30 min following the procedure as well as for the rest of the hospital stay. The left and right ventricle models of CARTO 3 were constructed in order to monitor the electrophysiological pattern of the patient following the procedure. It was observed that the 2 ablation points were in close proximity (Fig. 6).

## 3 Discussion

PVCs originating from aortic sinus cusps are encountered frequently and can be eliminated effectively by ablation. A limited number of case reports have demonstrated successful catheter ablation of PVCs that originate from the ROVT in patients with dextrocardia and situs inversus.^[1,2]^ However, a case of aortic sinus cusps–PVC–ablation using a 3D mapping system in dextrocardia has not been reported to date.

Catheter ablation is a challenging procedure in patients with dextrocardia, due to the complication of the interpretation of the intracardiac signals, the manipulation of the electrophysiology, and the unique nature of the ventricular anatomy.^[3]^ The identification of the accurate anatomy using the 3D electroanatomical mapping system combined with CT scanning or 3D image reconstruction may aid the understanding of the exact anatomical structures. In addition, the latter techniques can provide guidance for successful catheter access and enable the operator to conduct a safe catheter ablation procedure.

Furthermore, the mechanism of monomorphic ventricular arrhythmia is mainly due to cyclic adenosine monophosphate (c-AMP)-mediated calcium-dependent delayed after depolarizations and related to a arrhythmogenic cell focus firing and triggering induction mechanisms.^[4]^ Inflammation and oxidative stress play important role in ventricular arrhythmia,^[5]^ and they can significantly affect outcome of PVCs ablation, and they are important predictors of long-term efficacy of PVCs ablation.^[6]^ Higher inflammatory status may represent a chronic irritative mechanism, leading to subclinical alterations that affect ablation outcomes, and antioxidative therapies can reduce postablation recurrences.^[6,7]^ One study from Italy about patients with metabolic syndrome (MS) received PVCs ablation, they found that the patients with MS had significantly higher recurrence rate of PVCs due to higher inflammation status.^[6]^ The patient in our report was not a MS patient, because the inflammation and oxidative stress markers are normal at follow up (Table 1), so we did not apply any antioxidative therapies for this patient.

In conclusion, the present study suggests that the placement of the ECG electrodes in reversed positions combined with the 3D electroanatomical mapping system can lead to a successful catheter ablation for PVCs in patients with dextrocardia, provided careful attention is paid to the specific anatomical details.
